# CT radiomics prediction of CXCL9 expression and survival in ovarian cancer

**DOI:** 10.1186/s13048-023-01248-5

**Published:** 2023-08-30

**Authors:** Rui Gu, Siyi Tan, Yuping Xu, Donghui Pan, Ce Wang, Min Zhao, Jiajun Wang, Liwei Wu, Shaojie Zhao, Feng Wang, Min Yang

**Affiliations:** 1 School of Pharmacy, Nanjing Medical University, Nanjing, 211166 China; 2 Key Laboratory of Nuclear Medicine, Ministry of Health, Jiangsu Key Laboratory of Molecular Nuclear Medicine, Jiangsu Institute of Nuclear Medicine, Wuxi, 214063 China; 3 Department of Gynecology, Wuxi Maternity and Child Health Care Hospital, Wuxi School of Medicine, Jiangnan University, Wuxi, 214002 China; 4 Department of Gynecology, The Affiliated Wuxi Maternity and Child Health Care Hospital of Nanjing Medical University, Wuxi, 214000 China; 5 Department of Nuclear Medicine, Nanjing First Hospital, Nanjing Medical University, Nanjing, 210001 China

**Keywords:** Ovarian cancer, CXCL9, Radiomics, Prognosis, Overall survival

## Abstract

**Background:**

C-X-C motif chemokine ligand 9 (CXCL9), which is involved in the pathological processes of various human cancers, has become a hot topic in recent years. We developed a radiomic model to identify CXCL9 status in ovarian cancer (OC) and evaluated its prognostic significance.

**Methods:**

We analyzed enhanced CT scans, transcriptome sequencing data, and corresponding clinical characteristics of CXCL9 in OC using the TCIA and TCGA databases. We used the repeat least absolute shrinkage (LASSO) and recursive feature elimination(RFE) methods to determine radiomic features after extraction and normalization. We constructed a radiomic model for CXCL9 prediction based on logistic regression and internal tenfold cross-validation. Finally, a 60-month overall survival (OS) nomogram was established to analyze survival data based on Cox regression.

**Results:**

CXCL9 mRNA levels and several other genes involving in T-cell infiltration were significantly relevant to OS in OC patients. The radiomic score (rad_score) of our radiomic model was calculated based on the five features for CXCL9 prediction. The areas under receiver operating characteristic (ROC) curves (AUC-ROC) for the training cohort was 0.781, while that for the validation cohort was 0.743. Patients with a high rad_score had better overall survival (*P* < 0.001). In addition, calibration curves and decision curve analysis (DCA) showed good consistency between the prediction and actual observations, demonstrating the clinical utility of our model.

**Conclusion:**

In patients with OC, the radiomics signature(RS) of CT scans can distinguish the level of CXCL9 expression and predict prognosis, potentially fulfilling the ultimate purpose of precision medicine.

**Supplementary Information:**

The online version contains supplementary material available at 10.1186/s13048-023-01248-5.

## Background

Ovarian cancer (OC) has the highest mortality rate among all gynecological tumors, with no early symptoms, owing to the deep pelvic location of the ovaries and broad drug resistance. Despite advances in treatments including surgery, chemotherapy, targeted therapy, and immunotherapy, the overall survival (OS) rate of patients with OC remains low; five-year survival is less than 30% and a three-year recurrence rate is more than 70% [1, 2]. Furthermore, owing to high tumor heterogeneity, classic biomarkers and imaging indicators such as serum CA125 and transvaginal ultrasound are insufficient for monitoring therapy, which may lead to misdiagnosis or overtreatment [3, 4]. Therefore, new prognostic markers must be explored to provide individualized precision treatments.

In addition to malignant tumor cells, tumors contain normal cells, including immune cells, fibroblasts, and epithelial cells. The tumor immune microenvironment (TIME) is composed of these cells, and inflammatory immune cells act as the initial line of immune protection against pathogens [5, 6]. The mobilization of lymphocytes, a symptom of inflammation and a characteristic feature of malignancy, requires a multitude of cytokines and stimulating agents [7]. Chemokines, as a type of cytokines in the TIME, may be associated with patient outcomes. The CXC chemokine subfamily member CXCL9 encodes secreted proteins that play essential roles in disease processes, such as inflammation, immune regulation, tumor metastasis, and angiogenesis [8–10]. In addition to its two family members, CXCL10 and CXCL11, CXCL9 has been reported to enhance antitumor lymphocyte infiltration through its receptor CXCR3 in solid tumors, such as colorectal cancer, bladder cancer, gastric cancer, and uterine corpus endometrial carcinoma [11–14]. The same is true for ovarian cancer, where preclinical models have demonstrated a positive correlation between CXCL9 expression and T cell infiltration and overall survival [15–17]. In view of the promising clinical applications of CXCL9, recent clinical studies have focused on its role in diseases such as COVID-19, autoimmune diseases, and cancer [18–23]. In ovarian cancer, Au et al. indicated that high levels of CXCL9 are associated with an enhanced response to chemotherapy [24]. In addition, CXCL9 may be a reliable biomarker for predicting the immune checkpoint blockade (ICB) response in patients with OC due to CXCR3 chemokine activity being essential for effective immune checkpoint suppression [22, 23].

CXCL9 expression detected in the peripheral blood may not be representative of the tumor parenchyma. Given the remarkable tumor heterogeneity in OC and the impractical and invasive procedure of repeated biopsy, the whole tumor lesion and response to therapy are difficult to assess using conventional biopsies. Computed tomography (CT), which is widely used in clinical practice, is a common imaging method for OC diagnosis, treatment evaluation, and postoperative follow-up. Notably, rapid advances in artificial intelligence mean that radiomics, a high-throughput data mining approach that extracts massive image parameters, can now dynamically, noninvasively, and quantitatively assess the entire three-dimensional tumor [25, 26]. Previous reports have suggested that radiomics can be utilized not only in diagnosis for early OC, subtype classification, and lymph node metastasis, but also for the assessment of residual lesions, tumor heterogeneity, and TIME [27–32]. However, CXCL9 expression has not yet been predicted using radiomics in patients with OC.

Therefore, we developed a radiomic model using the TCGA and TCIA databases to predict CXCL9 expression in patients with OC and explored its prognostic value.

## Methods

### Data access

We extracted transcriptome sequencing data, enhanced computed tomography (CT) scans, and corresponding clinicopathological information from The Cancer Imaging Archive (TCIA, https://www.cancerimagingarchive.net/)database, as well as The Cancer Genome Atlas (TCGA, https://portal.gdc.cancer.gov/) database, to investigate the prognostic significance of CXCL9, build a radiomic model for predicting CXCL9 status in OC, and identify its prognostic worth.

For assessment of prognostic significance, several variables were included as covariates, such as chemotherapy, age, residual tumor disease, venous invasion, histological grade, lymphatic invasion, and International Federation of Gynecology and Obstetrics (FIGO) stage. The main outcome was OS. Patients with 1) non-primary OC and missing clinical data, such as OS, FIGO stage, and follow-up of < 30 days (prognostic value of CXCL9); 2) unqualified pre- or post-treatment CT scanning images (radiomics to predict CXCL9 expression); and 3) no OS and follow-up of < 30 days (prognostic value of radiomics) were excluded. Supplemental Table 1 presents detailed inclusion and exclusion criteria.

**Table 1:** Baseline characteristics between CXCL9-high and CXCL9-low groups

Variables	Total (*n* = 339)	Low (*n* = 189)	High (*n* = 150)	p
Age, n (%)				0.374
~ 59	175 (52)	93 (49)	82 (55)	
60 ~	164 (48)	96 (51)	68 (45)	
Chemotherapy, n (%)				0.925
NO	21 (6)	11 (6)	10 (7)	
YES	318 (94)	178 (94)	140 (93)	
Venous_invasion, n (%)				0.308
NO	32 (9)	20 (11)	12 (8)	
Unknown	248 (73)	141 (75)	107 (71)	
YES	59 (17)	28 (15)	31 (21)	
Lymphatic_invasion, n (%)				0.089
NO	40 (12)	25 (13)	15 (10)	
Unknown	208 (61)	122 (65)	86 (57)	
YES	91 (27)	42 (22)	49 (33)	
Tumor_residual_disease, n (%)				0.696
No Macroscopic disease	58 (17)	32 (17)	26 (17)	
1–10 mm	162 (48)	95 (50)	67 (45)	
10 mm ~	86 (25)	46 (24)	40 (27)	
Unknown	33 (10)	16 (8)	17 (11)	
Histologic_grade, n (%)				0.83
G1/G2	41 (12)	24 (13)	17 (11)	
G3/G4/GX	298 (88)	165 (87)	133 (89)	
FIGO_stage, n (%)				0.603
I/II	19 (6)	9 (5)	10 (7)	
III/IV	320 (94)	180 (95)	140 (93)	

### Survival analysis by CXCL9 expression and enrichment analysis of differential expressed genes (DEGs)

We extracted RNA-Seq data incorporating clinical information from TCGA and Gene-Tissue Expression (GTEx) from UCSC XENA using Xiantao online visualization toolset (https://www.xiantao.love/login). All eligible cases were classified as CXCL9-high or CXCL9-low group, according to their cutoff expression levels obtained by the R package “survminer”. RNA-seq expression data were downloaded, processed and reported as transcripts per million reads (TPM) by the Toil process [33], and were compared between samples after log2 transformation using the R package "ggplot2 [version 3.3.3]". Univariate analysis, followed by multivariate analysis, was introduced to estimate hazard ratios (HRs) with 95% confidence intervals (CI) for variables by means of COX proportional hazards model to report, for both subgroup analysis and interaction testing. Correlation analyses between CXCL9 levels and clinical characteristics were completed using Spearman’s rank correlation coefficient.

Differential immune gene expression of the CXCL9-high group from CXCL9-low group was analyzed using the Wilcoxon test. Immune cell infiltration in each sample was calculated using the CIBERSORTx database (https://cibersortx.stanford.edu/). Correlation analysis between CXCL9 expression and immune cell infiltration was completed based on the Spearman’s rank correlation coefficient. We analyzed the data using functional enrichment to confirm the functions of the potential targets. R package "clusterProfiler” was utilized to visualize the top ten significantly enriched terms from Gene Ontology (GO) analysis and the top thirty enriched pathways from Kyoto Encyclopedia of Genes and Genomes (KEGG) enrichment analysis.

### Developing radiomic models to determine CXCL9 expression levels in OC

Volumes of interest (VOIs) were created by manually tracing tumors on CT using 3D Slicer software (version 4.10.2) by an experienced radiologist in a double-blind manner. Another experienced radiologist performed accordingly in 10 randomly selected patients, to verify the results. We extracted 107 radiomic features (RFs) using an open source Python software package, PyRadiomics (https://pyradiomics.readthedocs.io/en/latest/) and conducted normalization, including resampling images with the same voxel size and Z-score standardization. We conducted a reliability evaluation of feature extraction and image segmentation using the intraclass correlation coefficient (ICC), and included RFs with an ICC of ≥ 0.8 in our study.

We used repeat (1,000 times) LASSO and RFE methods to screen RFs, and LR was applied to construct our radiomic model, in which the rad_score was used to predict CXCL9 expression.

We used ROC, precision-recall (PR) curves, AUC, and other diagnostic indices to assess discriminatory capacity, and DCA to estimate the clinical net benefit. Diagnostic indices included accuracy, sensitivity, specificity, positive predictive value (PPV), negative predictive value (NPV),and Brier Score. Calibration curves were used to assess the calibration of our model, and the Hosmer–Lemeshow goodness of fit statistic was used to evaluate the diagnostic model fit. We conducted an internal tenfold cross-validation to verify the proposed model.

### Prognostic relevance of of the radiomic model in patients with OC

The final radiomic model selection was performed using a stepwise selection approach with minimization of the Akaike information criterion (AIC). Patients were classified into two groups, high Rad_score and low Rad_score, on the basis of the probability threshold obtained using the R package “survminer”. Time-dependent ROC curves were used to assess the discrimination. Calibration curves were constructed to compare predicted and observed 60-month survival probabilities. A nomogram was developed to predict 60-month survival based on Cox regression and was assessed using DCA.

### Statistical analysis

Categorical variables were expressed as relative distribution frequencies (percentages), whereas continuous variables were expressed as mean ± standard deviation. Categorical variables between two groups were compared using the chi-square test, whereas continuous variables were compared using the Wilcoxon test. The DeLong test was used to assess statistical differences in the AUCs of the nomogram, Rad-score, and ROC curves. All data were statistically analyzed by R software. Statistical significance was set at *P* < 0.05, based on 2-tailed tests.

## Results

### Clinical significance of CXCL9

In total, 339 patients were included in the TCGA-OC project and divided into CXCL9-high (*n* = 150) and CXCL9-low (*n *= 189) groups with a cut-off expression level of 2.829. Table 1 summarizes the baseline patient characteristics of the TCGA-OV database. The covariates represented no significant difference between the two groups (*P* > 0.05).

**Fig. 1:**
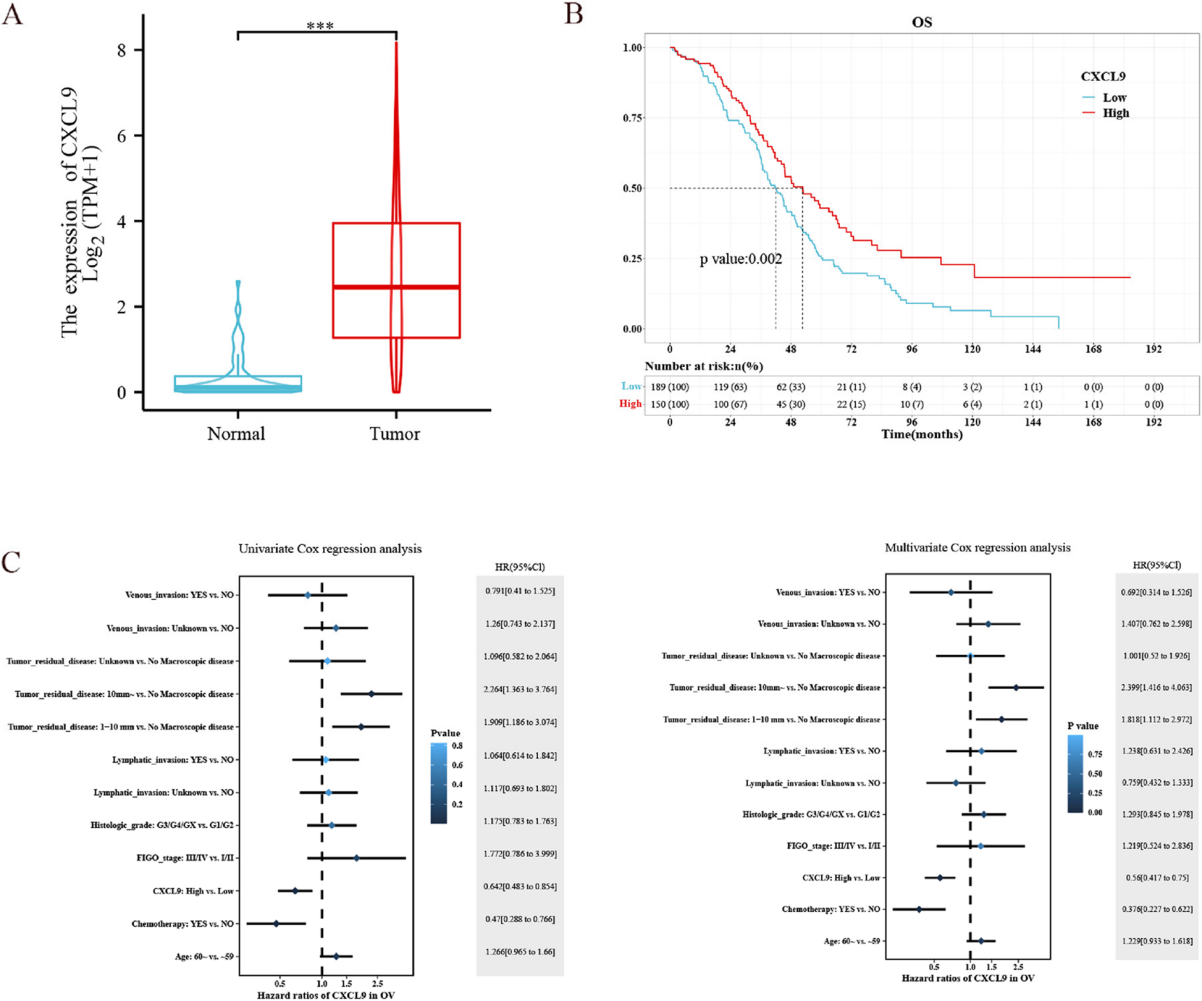
CXCL9 expression comparison, survival analysis, Cox regression analysis of TCGA-OV cohort. **A** The expression level of CXCL9 in OC tissues was signifcantly higher than that in normal tissues; **B** The Kaplan–Meier curve showed that high CXCL9 expression was significantly associated with improvement in patients’ OS; **C** Univariate and multivariate COX regression demonstrated the protective impact of high CXCL9 expression on the OS.* *P* < 0.05, ** *P* < 0.01, *** *P* < 0.001

**Fig. 2:**
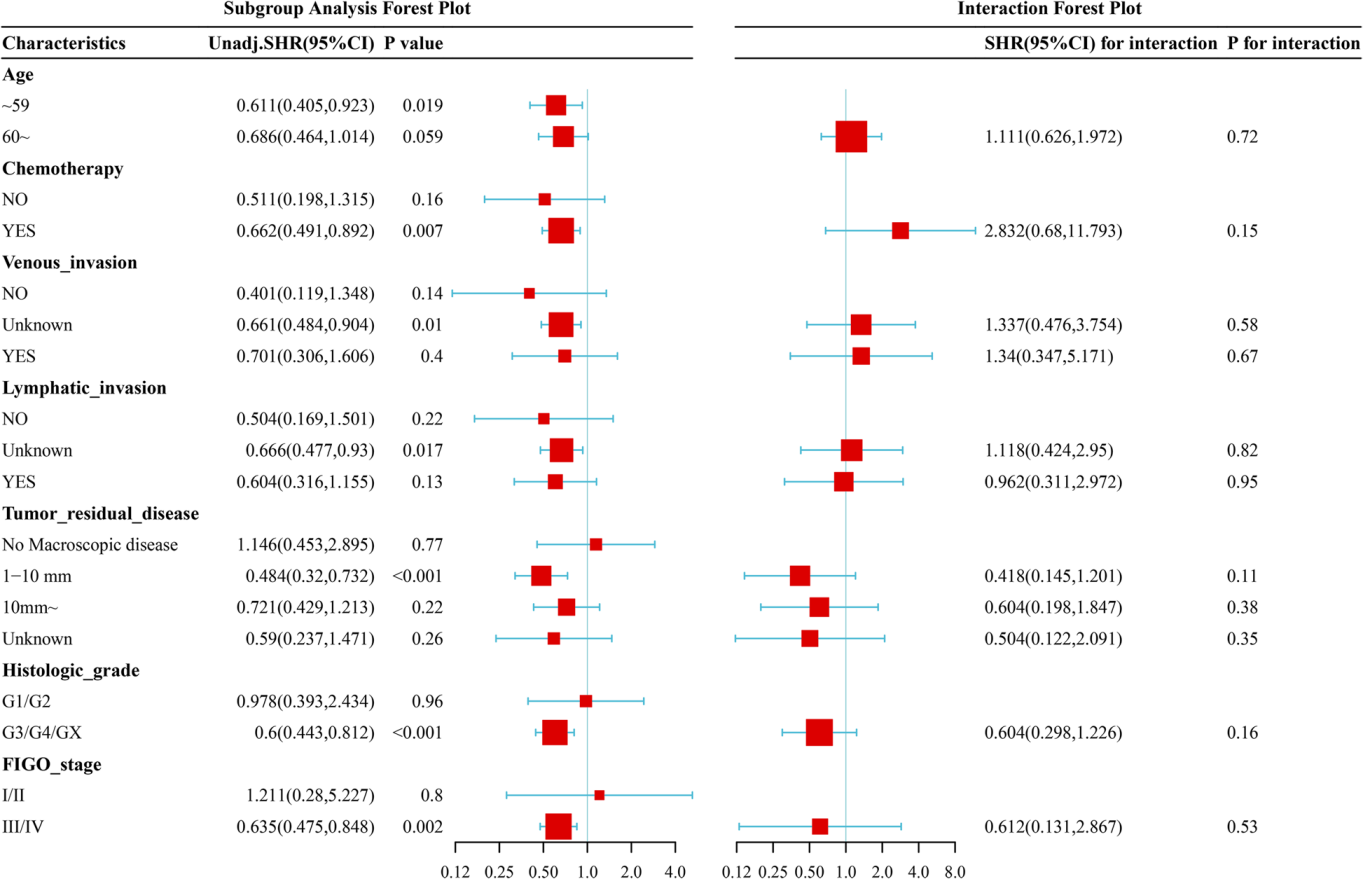
Univariate subgroup analysis and interaction test. High CXCL9 expression level was protective in OC patients aged < 60 years or accepted chemotherapy. Age and CXCL9 expression, chemotherapy and CXCL9 expression had no significant interaction effects on the OS

**Fig. 3:**
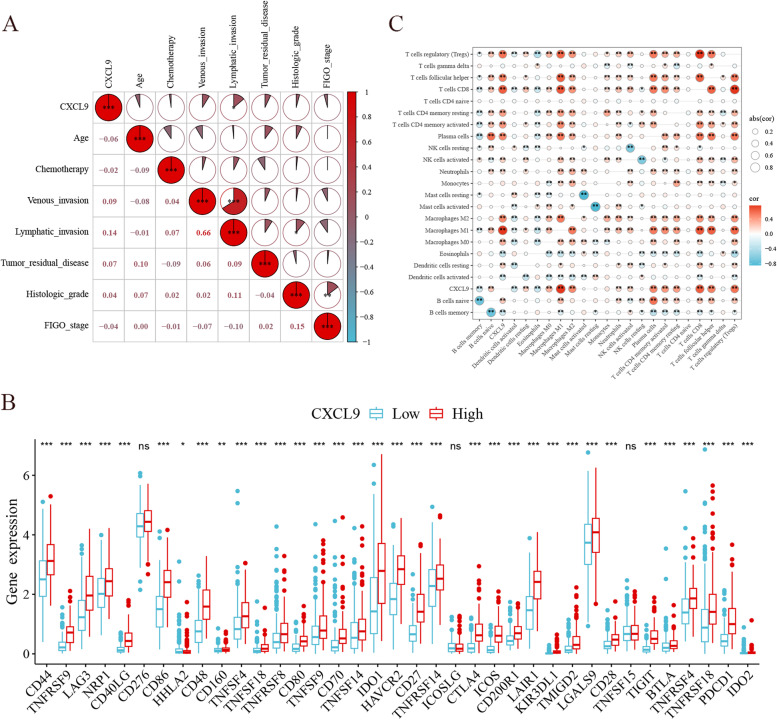
Relationship analysis between CXCL9 expression level and clinical characteristics, immuno-infiltrations analysis. **A** High CXCL9 expression was positively associated with the lymphatic invasion; **B** The expression levels of CD44, TNFRSF9 and LAG3 were significantly increased in the CXCL9-high group;**C** CXCL9 was positively correlated with CD8 + T cell infiltration abundance.* *P* < 0.05, ** *P* < 0.01, *** *P* < 0.001

CXCL9 was upregulated in 427 TCGA-OV tumor samples compared to 88 GTEx normal ovary samples(*P* < 0.001, Fig. 1A). As shown in Fig. 1B, the Kaplan–Meier curve indicated that the median survival time was 41.97 months in the CXCL9-low group and 52.63 months in the CXCL9-high group, suggesting high CXCL9 expression was positively associated with survival for patients with OC (*P* = 0.002). Univariate and multivariate COX regression analyses verified the positive effect of CXCL9 on overall survival. After the subgroup analysis via univariate model and interaction test via likelihood ratio test, high CXCL9 expression showed a protective and positive role in OC patients who received chemotherapy and in those aged < 60 years. CXCL9 and age, CXCL9 and chemotherapy did not interact to show a significant impact on OS, as shown in Fig. 2. High CXCL9 expression was associated with enhanced lymphatic invasion (Fig. 3A).

The expression levels of CD44, TNFRSF9, LAG3 and CD8 + T cell infiltration abundance were positively correlated with the increased expression of CXCL9 (Fig. 3B,3C). Based on GO enrichment analysis, the DEGs screened in this study were significantly enriched in immunoglobulin receptor binding and immunoglobulin complexes (Supplemental Fig. 1A). KEGG pathways analysis of DEGs of CXCL9 also showed an enrichment of chemokines and Th17 cell differentiation (Supplemental Fig. 1B).

### Radiomic model construction and evaluation

Finally, 57 patients with OC were enrolled from the TCIA-CT database. Among the extracted radiomic features, 91.6% had an ICC > 0.8, 6.5% had an ICC between 0.5 and 0.79, and 1.9% had an ICC < 0.5. After filtration, 98 out of 107 radiomic features (91.6%) with ICC ≥ 0.8 were included for further analysis.

### LASSO-LR radiomic model

**Fig. 4:**
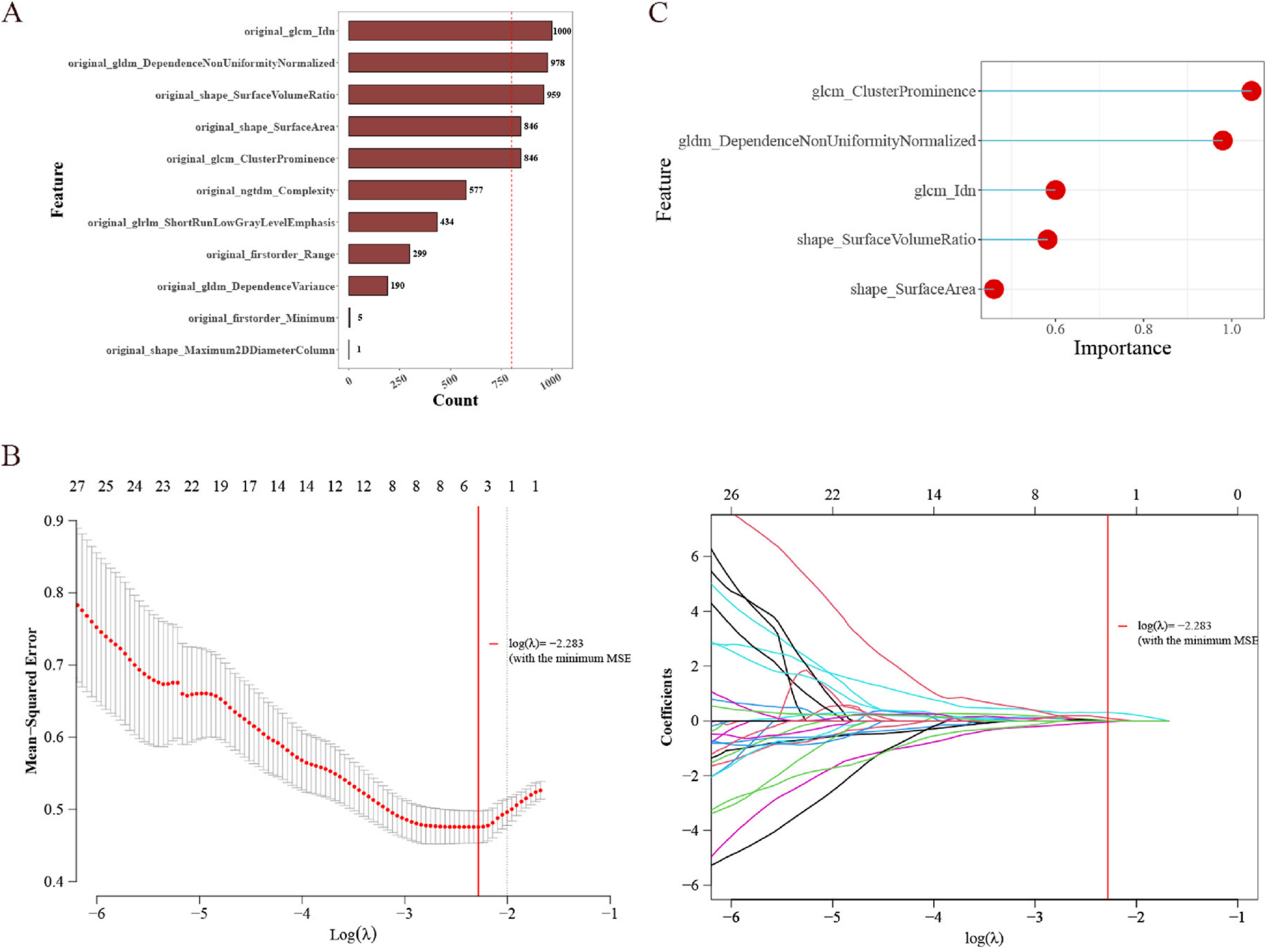
Feature selection of the radiomic model.**A** Features histogram;**B** Feature reduction in the repeat LASSO logistic regression model;**C** Five optimal features:glcm_Idn, gldm_DependenceNonUniformityNormalized, shape_SurfaceVolumeRatio, glcm_ClusterProminence and shape_SurfaceArea

A histogram was plotted for the features with most counts (Fig. 4A). Five features remained after LASSO screening (Fig. 4B). Figure 4C illustrates these features and their significance.

**Fig. 5:**
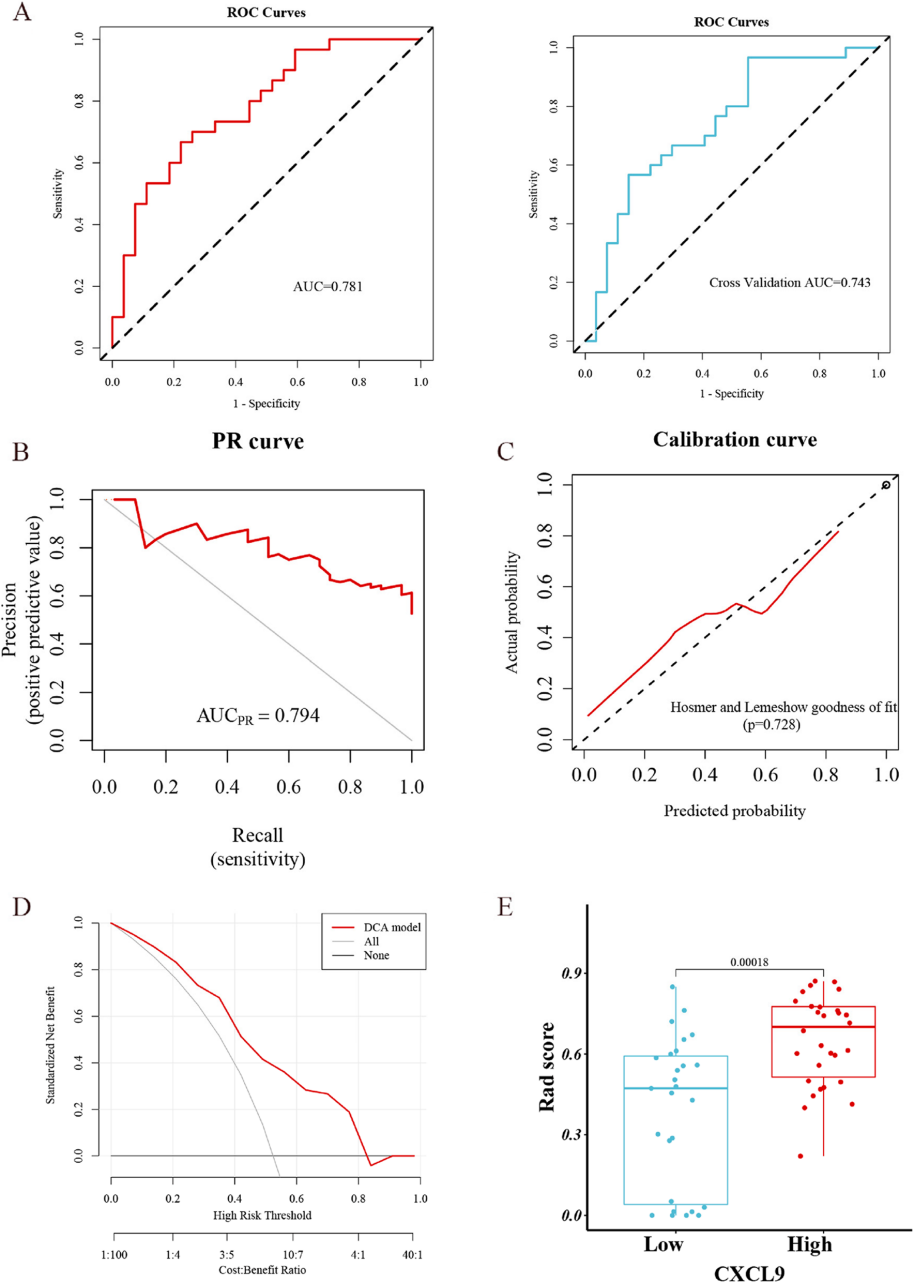
Evaluation of the radiomic model for prediction of CXCL9 expression: **A** Receiver operating characteristic(ROC) curves in the training set and the validation set; **B** Precision recall(PR) curves in the training set;**C** calibration curves;**D** Decision curve analysis(DCA); **E** Box plots of predicted probabilities in CXCL9-high and CXCL9-low groups

In the training sets, the accuracy, sensitivity, specificity, PPV, NPV, and Brier score were 0.719,0.667,0.778,0.769,0.677 and 0.184, respectively; in the validation cohort, the accuracy, sensitivity, specificity, PPV, NPV, and Brier score were 0.702,0.567,0.852,0.81,0.639 and 0.203, respectively. In the training sets, the ROC curve achieved AUC values of 0.781, and the PR curve achieved AUC values of 0.794; in the validation sets, the ROC curve achieved AUC values of 0.743. The DeLong test between the cross-validation AUCs did not show a significant difference between the results, indicating a good model fit (Fig. 5A, B).

Calibration curves derived from the Hosmer–Lemeshow test showed that the LASSO-LR predictive model fitted well with the actual gene expression. As shown in the DCA, the LASSO-LR model yielded a threshold probability of 0–0.82, resulting as the highest net benefit, when compared with all treatments and no treatment schemes. High CXCL9 level was with greater discrimination in their probability estimates of the CXCL9-high group than in other groups (Fig. 5C, D & E).

### RFE-LR radiomic model

Three features remained after the RFE screening (Supplemental Fig. 3). The RFE-LR radiomic model showed a good prediction effect with an AUC-ROC of 0.765 and 0.759 in the training and validation datasets, respectively, and DCA provided evidence of its high clinical utility. The results of predictive probability of high CXCL9 expression suggested an increased positive predicted probability of participants to be a high producer of CXCL9(*P* < 0.001,Supplemental Fig. 4).

### LASSO and RFE feature intersection-LR radiomic model

Supplemental Fig. 5 presents two common features between the LASSO and RFE methods and their significance. The intersection-LR radiomics model showed a good prediction effect with an AUC-ROC of 0.723 and 0.715 in the training and validation datasets, respectively, and the DCA provided evidence of its high clinical utility. High CXCL9 expression was with greater discrimination in their probability estimates in the CXCL9-high group (*P* < 0.001,Supplemental Fig. 6).

All three radiomics models described above exhibited good predictive efficacy, and the comparison of AUCs using the DeLong test revealed no statistically significant differences among the three models. However, considering the AUC and PR curves of each model, the LASSO-LR model was better; thus, the predicted value of the LASSO-LR model was used for subsequent prognostic analysis (Supplemental Table 4).

### Prognostic value of the LASSO-LR radiomic model in patients with OC

Ultimately, survival analysis included 57 patients from TCGA-OC divided into CXCL9-high (*n* = 46) and CXCL9-low (*n* = 11) groups, with a Rad_score cut-off expression level of 0.302. The covariates were not significantly different between the two groups (*P* > 0.05,Table 2).

**Table 2:** Baseline characteristics between Rad_score-high and Rad_score-low groups

Variables	Total (*n* = 57)	Low (*n* = 11)	High (*n* = 46)	p
Age, n (%)				0.948
~ 59	28 (49.1)	6 (54.5)	22 (47.8)	
60 ~	29 (50.9)	5 (45.5)	24 (52.2)	
Chemotherapy, n (%)				1
YES	57 (100)	11 (100)	46 (100)	
Venous_invasion, n (%)				0.87
NO	5 (8.8)	1 (9.1)	4 (8.7)	
Unknown	39 (68.4)	7 (63.6)	32 (69.6)	
YES	13 (22.8)	3 (27.3)	10 (21.7)	
Lymphatic_invasion, n (%)				0.682
NO	5 (8.8)	1 (9.1)	4 (8.7)	
Unknown	33 (57.9)	5 (45.5)	28 (60.9)	
YES	19 (33.3)	5 (45.5)	14 (30.4)	
Tumor_residual_disease, n (%)				0.635
1–10 mm	31 (54.4)	8 (72.7)	23 (50)	
10 mm ~	12 (21.1)	1 (9.1)	11 (23.9)	
No Macroscopic disease	9 (15.8)	1 (9.1)	8 (17.4)	
Unknown	5 (8.8)	1 (9.1)	4 (8.7)	
Histologic_grade, n (%)				1
G1/G2	5 (8.8)	1 (9.1)	4 (8.7)	
G3/G4/GX	52 (91.2)	10 (90.9)	42 (91.3)	
FIGO_stage, n (%)				0.244
I/II	5 (8.8)	2 (18.2)	3 (6.5)	
III/IV	52 (91.2)	9 (81.8)	43 (93.5)	
OS, n (%)				0.201
Alive	28 (49.1)	3 (27.3)	25 (54.3)	
Dead	29 (50.9)	8 (72.7)	21 (45.7)	
OS.time, Mean ± SD	42.9 ± 28.59	40.42 ± 21.11	43.49 ± 30.28	0.697

**Fig. 6:**
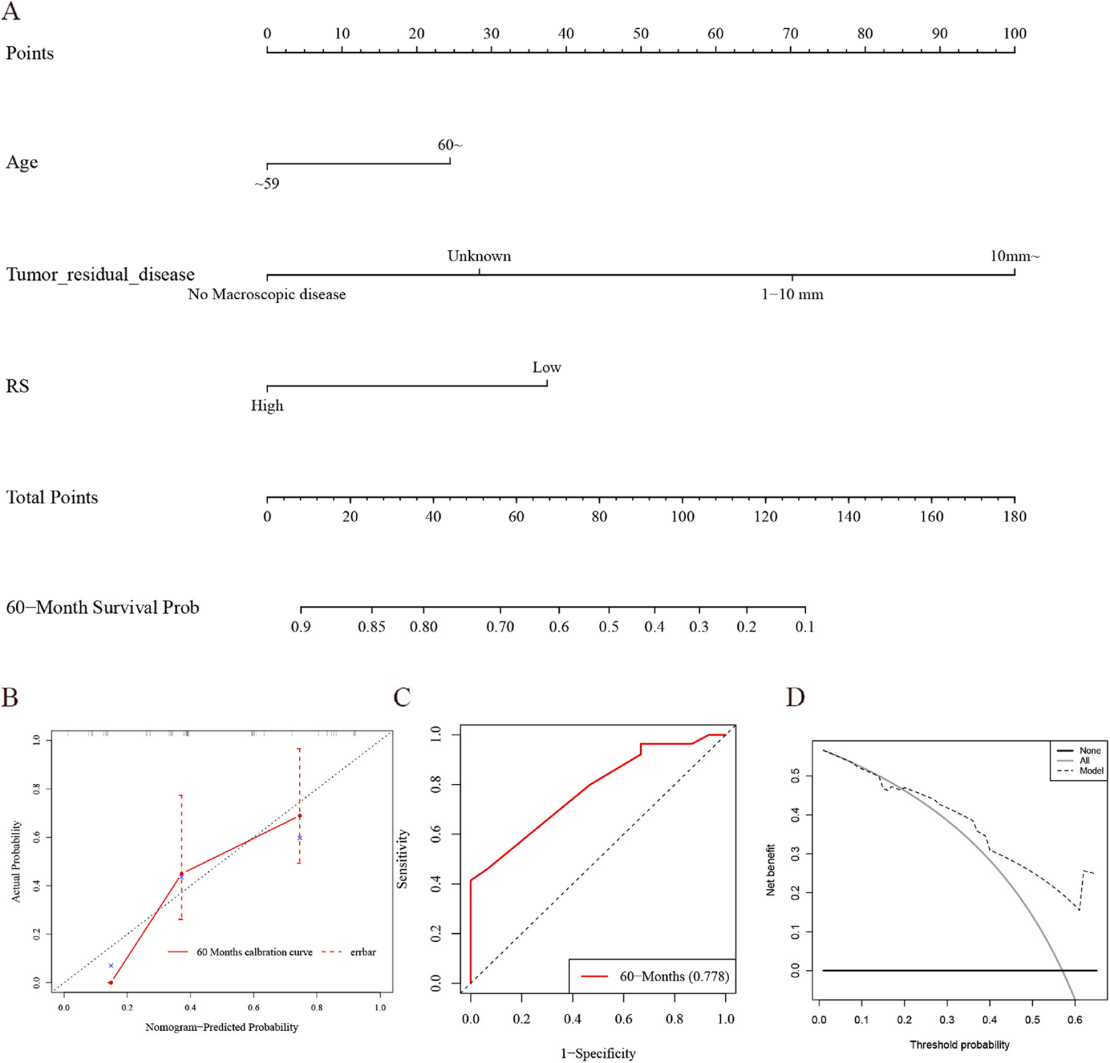
Nomogram and model evaluation. **A** Creation of the nomogram to predict the overall survival of a patient with ovarian cancer. **B** Calibration curves of the risk score; **C** The time-dependent ROC of the risk score; **D** DCA

The predicted LASSO-LR model was visualized in the form of a new nomogram. Each factor had a score on a point scale, such as age, tumor _residual_disease, and RS (Fig. 6A). Prognostic probability at each time point was estimated by drawing a straight line. In the calibration curves, the calibration of OS probability was assessed by comparing the observed 60-month OS probabilities with the predicted OS probabilities. As is known, the prediction falls along a 45-degree diagonal, close to the ideal diagonal, in a perfectly calibrated model. As shown in Fig. 6B, the 60-month survival rate predicted using the nomogram was almost identical to the actual 60-month survival rate. As shown in Fig. 6C (ROC curves), the AUC for the 60-month survival rate was 0.778. DCA (60 months) showed high clinical utility of the model in the risk threshold range from 0.2 to 0.65 (Fig. 6D).

## Discussion

We built a radiomic model for the prediction of ovarian cancer prognosis based on molecular biomarkers, computed tomography (CT), and clinical information. We concluded that: (1) CXCL9 considerably influenced OS in patients with OC (HR = 0.56, 95% CI: 0.417–0.75, *P* < 0.001), (2) our prediction model displayed promising predictive performance with an AUC-ROC of 0.781 (95% CI: 0.662–0.901), and (3) the prediction of the 60-month survival rate by radiomics-based nomogram matched the actual observational data well with an AUC-ROC of 0.778.

Despite significant advances in cytoreductive surgery and systemic therapies, the survival rates for ovarian cancer are still vastly variable, owing to the considerable heterogeneity. Maximum tumor resection is believed to improve the prognosis of patients with ovarian cancer. Furthermore, for patients in advanced stages of the disease, aggressive treatment does not significantly extend patient survival, but reduces the quality of life [3]. Thus, additional prognostic information enables a better clinical decision prior to treatment. Increasing evidence has shown that overexpression of CXCL9 mediates the recruitment of tumor-targeted CXCR3 + T cells and natural killer (NK) cells in various solid cancers, and thereby suppresses tumor growth [8]. Studies on OC, although limited, have indicated that significant increases in CXCL9 levels are strong and independent predictors of improved survival in patients with OC [16, 22–24]. Our results agree well with those of previous studies and demonstrate the importance of CXCL9 in the survival of patients with OC. Specifically, parameters including age, tumor histological grade, and lymphatic invasion were included in both the univariate and multivariate Cox regression analyses, and the results revealed that high CXCL9 expression levels were protective for patient survival (HR = 0.56, 95% CI: 0.417–0.75, *P* < 0.001).

Radiomics is a rapidly advancing quantitative technique that attempts to capture tumor characteristics using advanced imaging features. Numerous studies have shown that radiomics can reveal tumor heterogeneity and the underlying genomic and biological characteristics [26]. The same is true for ovarian cancer, favorable radiomics applications in OC like early diagnosis, subtype classification, treatment response prediction, lymph node metastasis, and survival [29–32], raised the possibility of radiomics uncovering the CXCL9 status in OC patients.Therefore, we aimed to apply radiomics to the prediction of OC survival using CT features extracted from pretreatment images of primary tumors. So far as we know, the current study first report a radiomic model to noninvasively predicting CXCL9 expression in OC. The results revealed that the survival rate in the high rad_score group was consistently higher (*P* < 0.05). Considering the previous results, we believe that predicting CXCL9 expression levels based on radiomics would be helpful for clinical decision-making and individualized treatment.

Recently, deep learning radiomics integrated with RNA-Seq microenvironmental analysis has provided powerful insights into the molecular mechanisms of cancer, and contributed to survival prediction. Zhao [34] conducted a radiomics study to predict the Epstein-Barr virus status in gastric cancer, and the predictive power of the model was excellent in both the training and validation cohorts, which achieved AUCs of 0.919 and 0.939, respectively. Lu [35] used a radiomic model to predict EGFR status in lung cancer, and the prediction model using different features achieved AUCs of 0.68, 0.67, and 0.69. Moreover, an increasing number of researchers are investigating accurate and cost-effective methods to assess immune biomarkers with prognostic value in OC, among which radiomic models are becoming a trend for future studies. For example, Wan [31] built a radiomic model for C–C motif chemokine receptor type 5 (CCR5) status and OC survival analysis, which yielded an AUCs of 0.770. Gao’s radiomic model for PD-1 and OC survival analysis yielded AUCs of 0.810. Our predictive model for CXCL9 status consistently performed well in both the training and validation cohorts and achieved AUCs of 0.781 and 0.743, respectively. GLCM and GLDM textures are well-known high-order radiomic features that are highly correlated with tumorgrades [36]. In this study, we derived 5 radiomic features, among which GLCM and GLDM also played a significant role in describing the spatial relationships between pixels and distinctive heterogeneity, and reflected the expression levels of CXCL9. We further assessed the calibration ability and clinical efficacy of our model in predicting CXCL9 status in both the training and validation groups, and the results were consistent with other studies that demonstrated that radiomic models performed well in the survival prediction of OC patients [37].

Recently, some researchers have used CT texture analysis to predict treatment response and prognosis in patients with hepatic cancer [38] and glioblastoma [39]. Preoperative enhanced CT texture analysis helps predict complete response to treatment. However, correlating individual texture features with complex tumor biological processes remains a challenge because it is not possible to fully exploit meaningful clinical information for comprehensive analysis, nor to validate the reliability of the results with internal or external data. Therefore, it is common to construct multifactorial radiomics models to evaluate clinical outcomes [40]. In this study, we developed prognostic models using integrated clinical-radiogenomic information to analyze survival in patients with OC and demonstrated that RFs combined with clinical features can improve the accuracy of individual clinical decision-making, and thus has a high potential for evidence-based clinical translation for OC management.

However, given the exploratory nature of this retrospective study, future studies are required to validate the findings in this study. The limited number of cases is one of the main factors restricting our research from reaching reliable conclusions. Although tenfold internal cross-validation was used for the evaluation of signature model, lack of external cohort from OC patients is another limitation of the present study. In addition, radiomics studies have generally demonstrated predictive ability in cancers, but the sensitivity and specificity of the predictions were poorer using different models and radiological signatures. Additionally, free datasets can vary in CT image quality and show high imbalances, especially in the ratio of high versus low CXCL9 levels. Accordingly, larger cohort data from multiple centers may provide a solution for future research and result in practical applications.

## Conclusions

In conclusion, our model can be used to evaluate the prognostic risk in patients with OC. The radiomics-based nomogram, which incorporates clinical and CT characteristics, can predict CXCL9 status in a noninvasive manner, potentially fulfilling the ultimate purpose of precision medicine for ovarian cancer.

### Supplementary Information

**Additional file 1: Supplemental Table 1.** Inclusion and exclusion criteria for samples. **Supplemental Table 2.** Intraclass correlation efficient(ICC) evaluation. **Supplemental Table 3.** Formulas of the three radiomic models. **Supplemental Fig. 1.** GO and KEGG enrichment analysis of the CXCL9. **Supplemental Fig. 2.** Image processing and feature extraction. Supplemental Fig. 3. Feature selection of the RFE-LR radiomic model. **Supplemental Fig. 4.** Assessment of the RFE-LR radiomic model for predicting expression level of CXCL9. **Supplemental Fig. 5.** Feature selection of the intersection-LR radiomic model. **Supplemental Fig. 6.** Assessment of the intersection-LR radiomic model for level prediction of CXCL9. **Supplemental Table 4.** Comparison of the AUC values between the radiomic models.

## Data Availability

All data generated or analysed during this study are included in this published article and its supplementary information files.The datasets for this study were downloaded from TCIA (https://www.cancerimagingarchive.net/) and TCGA (https://portal.gdc.cancer.gov/) database.
