# Supplementary material for: CT radiomics prediction of CXCL9 expression and survival in ovarian cancer
Source: J Ovarian Res. 2023 Aug 30;16:180. doi: 10.1186/s13048-023-01248-5 (PMC10466849; doi:10.1186/s13048-023-01248-5)
Supplement: Supplementary file 1 — Additional file 1: Supplemental Table 1. Inclusion and exclusion criteria for samples. Supplemental Table 2. Intraclass correlation efficient(ICC) evaluation. Supplemental Table 3. Formulas of the three radiomic models. Supplemental Fig. 1. GO and KEGG enrichment analysis of the CXCL9. Supplemental Fig. 2. Image processing and feature extraction. Supplemental Fig. 3. Feature selection of the RFE-LR radiomic model. Supplemental Fig. 4. Assessment of the RFE-LR radiomic model for predicting expression level of CXCL9. Supplemental Fig. 5. Feature selection of the intersection-LR radiomic model. Supplemental Fig. 6. Assessment of the intersection-LR radiomic model for level prediction of CXCL9. Supplemental Table 4. Comparison of the AUC values between the radiomic models. [file 13048_2023_1248_MOESM1_ESM.docx]

**Supplementary material**

**Supplemental Table 1: Inclusion and exclusion criteria for samples**

| **TCGA-OV clinical data** | Excluded samples | Remaining samples |
| --- | --- | --- |
| Total samples | - | 587 |
| Screening: primary untreated ovarian cancer | 5 | 582 |
| Excluded: OS/OS.time=NA | 5 | 577 |
| Excluded: OS.time＜30 days | 12 | 565 |
| Excluded:FIGO_stage/Histologic_grade=NA | 5 | 560 |
| Screening: primary solid tumors with RNA-seq | 221 | **339** |
| **TCIA-OV CT** |  |  |
| Total samples | - | 143 |
| Excluded image：poor quality | 52 | 91 |
| Intersection of TCGA genomic and clinical data | 34 | **57** |

TCGA: The Cancer Genome Atlas; OC: ovarian cancer; OS: overall survival; TCIA: Cancer Imaging Archive; CT: computed tomography

**Supplemental Table 2: Intraclass correlation efficient(ICC) evaluation**

|  | ICC≥0.8 | 0.5≤ICC<0.8 | ICC<0.5 | ICC_Mean | ICC_Median |
| --- | --- | --- | --- | --- | --- |
| Percentage | 0.916 | 0.065 | 0.019 | 0.946 | 0.985 |
| Number | 98 | 7 | 2 | NA | NA |

**Supplemental Table 3: Formulas of the three radiomic models**

| **RFE-LR radiomic model** | Estimate | Std. Error | z value | Pr(>\|z\|) |
| --- | --- | --- | --- | --- |
| (Intercept) | -0.729 | 0.970 | -0.752 | 0.452 |
| original_gldm_DependenceNonUniformityNormalized | -3.403 | 3.349 | -1.016 | 0.309 |
| original_shape_SurfaceVolumeRatio | 0.516 | 0.360 | 1.435 | 0.151 |
| original_gldm_SmallDependenceHighGrayLevelEmphasis | -1.305 | 0.950 | -1.374 | 0.169 |

| **LASSO-LR radiomic model** | Estimate | Std. Error | z value | Pr(>\|z\|) |
| --- | --- | --- | --- | --- |
| (Intercept) | -0.925 | 1.221 | -0.757 | 0.449 |
| original_shape_SurfaceArea | -0.258 | 0.562 | -0.460 | 0.645 |
| original_shape_SurfaceVolumeRatio | 0.296 | 0.510 | 0.582 | 0.561 |
| original_glcm_ClusterProminence | -0.859 | 0.822 | -1.045 | 0.296 |
| original_glcm_Idn | -0.270 | 0.451 | -0.600 | 0.548 |
| original_gldm_DependenceNonUniformityNormalized | -4.230 | 4.317 | -0.980 | 0.327 |

| **intersection-LR radiomic model** | Estimate | Std. Error | z value | Pr(>\|z\|) |
| --- | --- | --- | --- | --- |
| (Intercept) | -0.469 | 0.910 | -0.516 | 0.606 |
| original_gldm_DependenceNonUniformityNormalized | -2.944 | 3.175 | -0.927 | 0.354 |
| original_shape_SurfaceVolumeRatio | 0.717 | 0.363 | 1.977 | 0.048 |

**Supplemental Fig. 1: GO and KEGG enrichment analysis of the CXCL9**

**
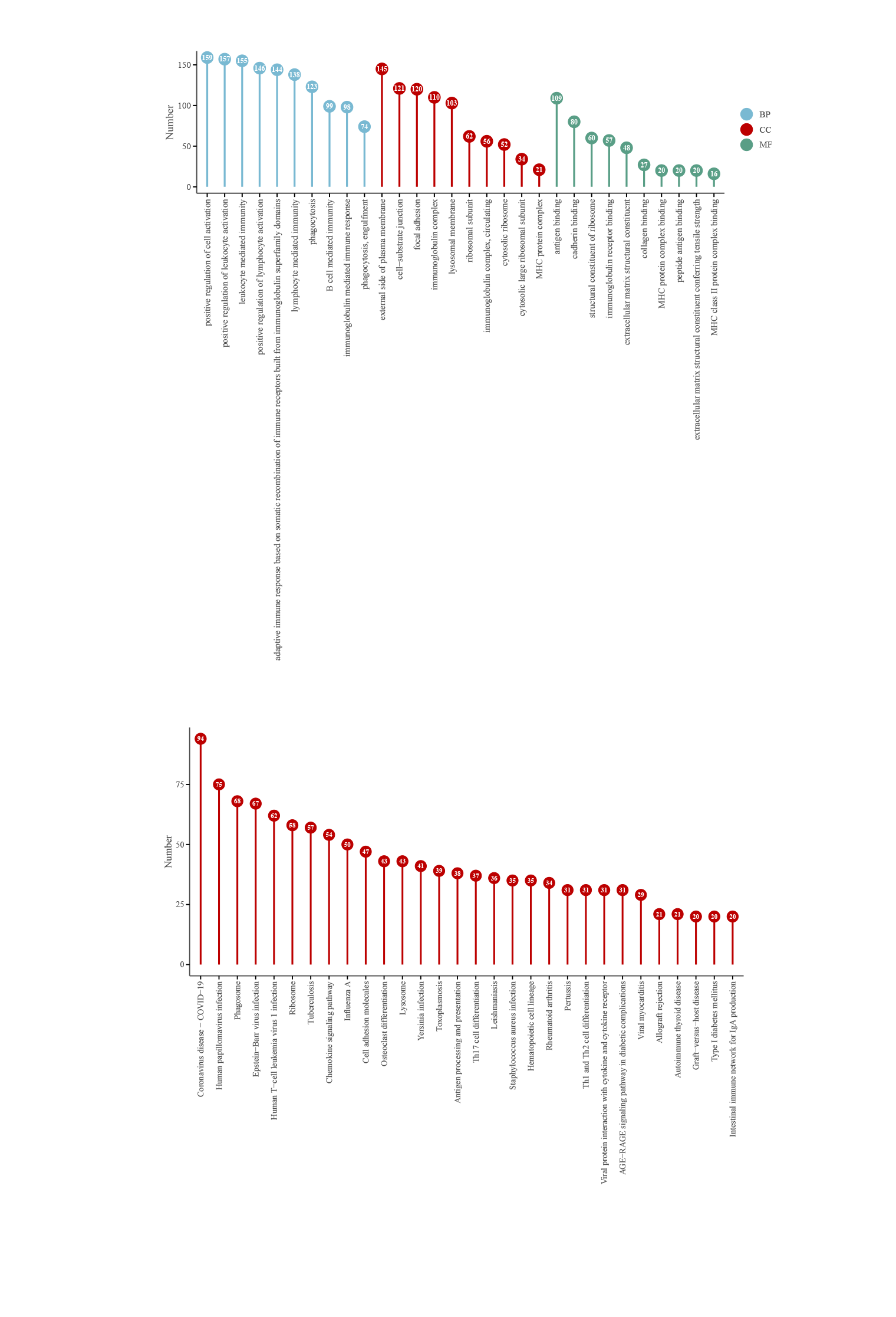
**

**Supplemental Fig. 2: Image processing and feature extraction**

**
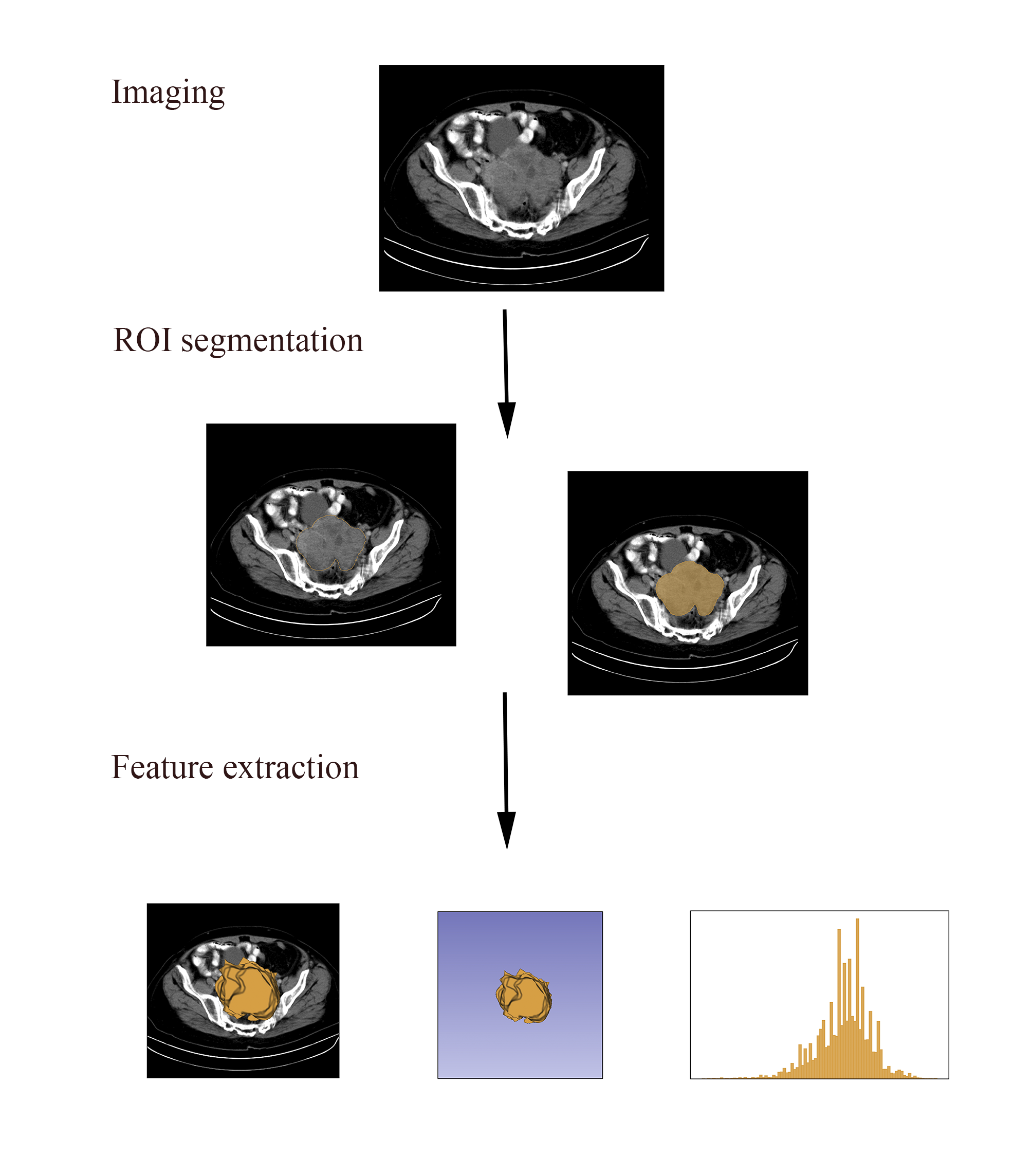
**

**Supplemental Fig. 3：Feature selection of the RFE-LR radiomic model.** Three features remained: gldm_DependenceNonUniformityNormalized, shape_SurfaceVolumeRatio and gldm_SmallDependenceHighGrayLevelEmphasis.


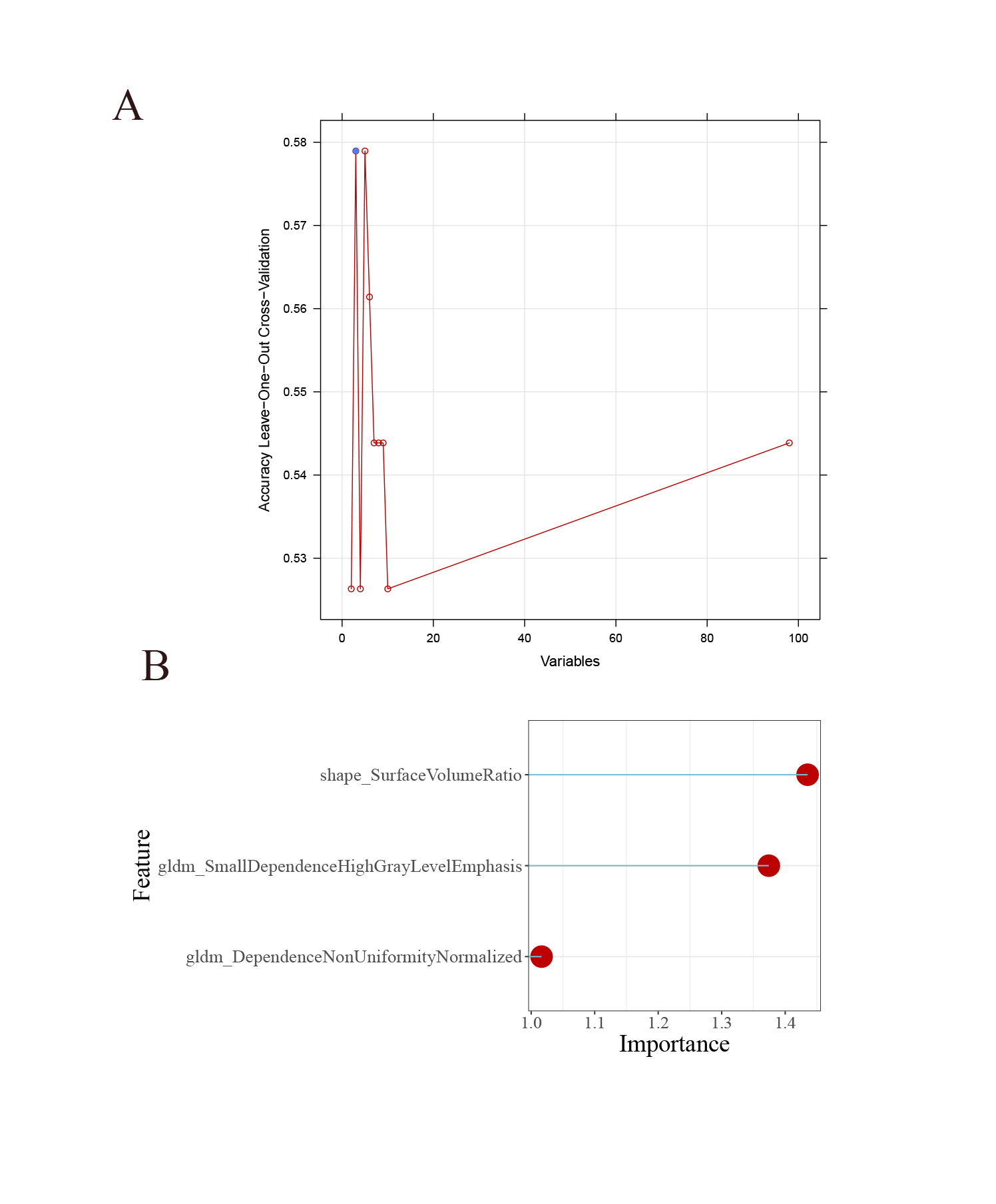


**Supplemental Fig. 4：Assessment of the RFE-LR radiomic model for predicting expression levels of CXCL9.** The training sets presented an accuracy of 0.702, a sensitivity of 0.467 , a specificity of 0.963, PPV of 0.933 , NPV of 0.619, and Brier score of 0.190; The validation cohort showed an accuracy of 0.702, a sensitivity of 0.467, a specificity of 0.963, PPV of 0.933, NPV of 0.619, and Brier score of 0.206. In the training sets, the ROC curve(**A**) showed AUC values of 0.765 (95% CI: 0.642-0.889), and the PR curve (**C**) showed AUC values of 0.773. In the validation cohort, the ROC curve (**B**) showed AUC values of 0.759 (95% CI: 0.634-0.885). The DeLong test between the cross-validation AUCs did not show a significant difference between the results, indicating a good model fit (P = 0.945). Calibration curves (**D**) calculated and plotted via Hosmer-Lemeshow test demonstrated that our predictive model showed a good consistency with the actual gene levels (P = 0.893). As shown in the DCA(**E**), the RFE-LR radiomic model was with the maximum net benefit (a threshold probability of 0-0.79). High CXCL9 expression was with greater discrimination in their probability estimates in the CXCL9-high group. (P < 0.001) (**F**).
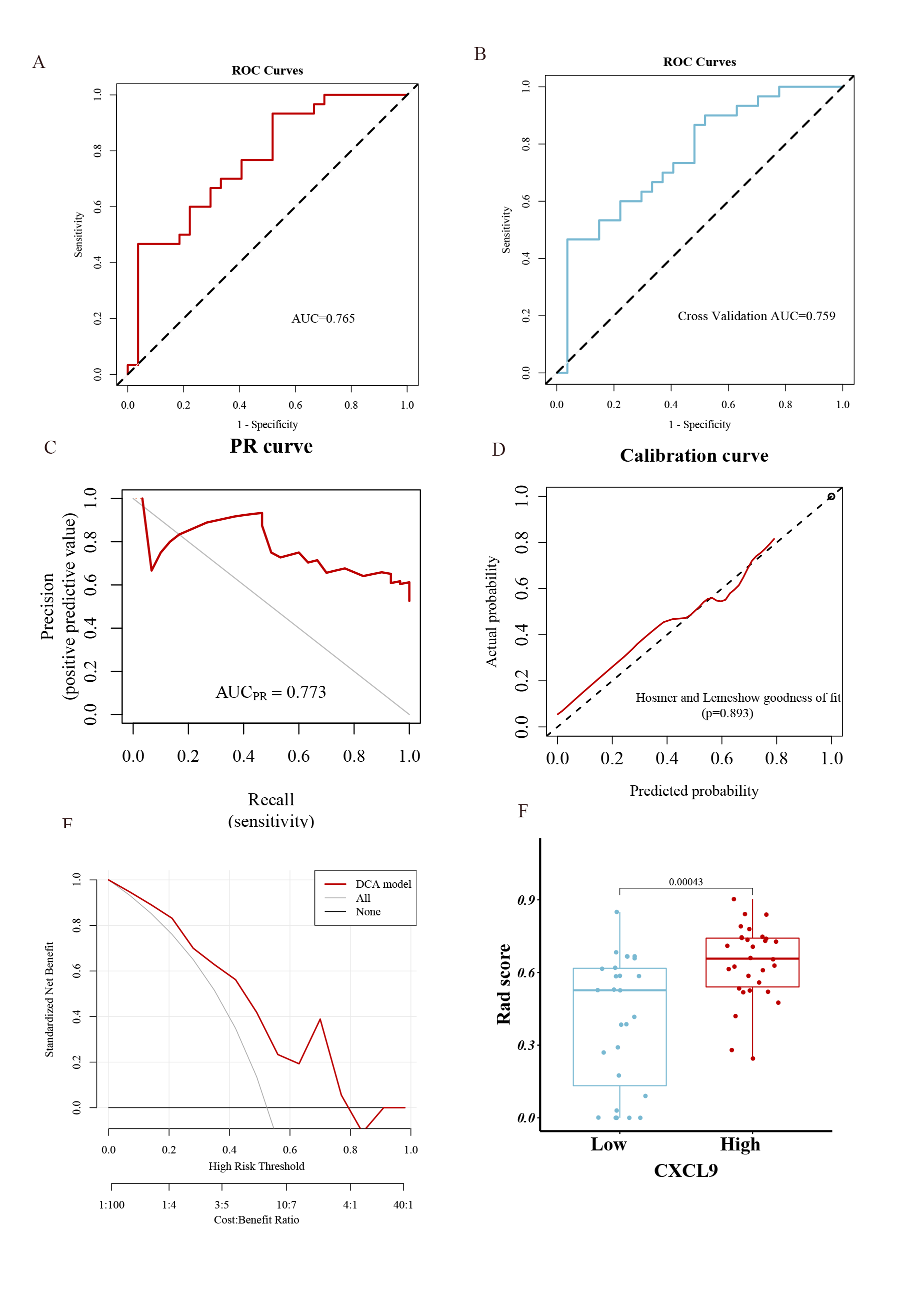


**Supplemental Fig. 5：Feature selection of the intersection-LR radiomic model.** Two common features between the LASSO and RFE methods were gldm_DependenceNonUniformityNormalized and shape_SurfaceVolumeRatio.

**
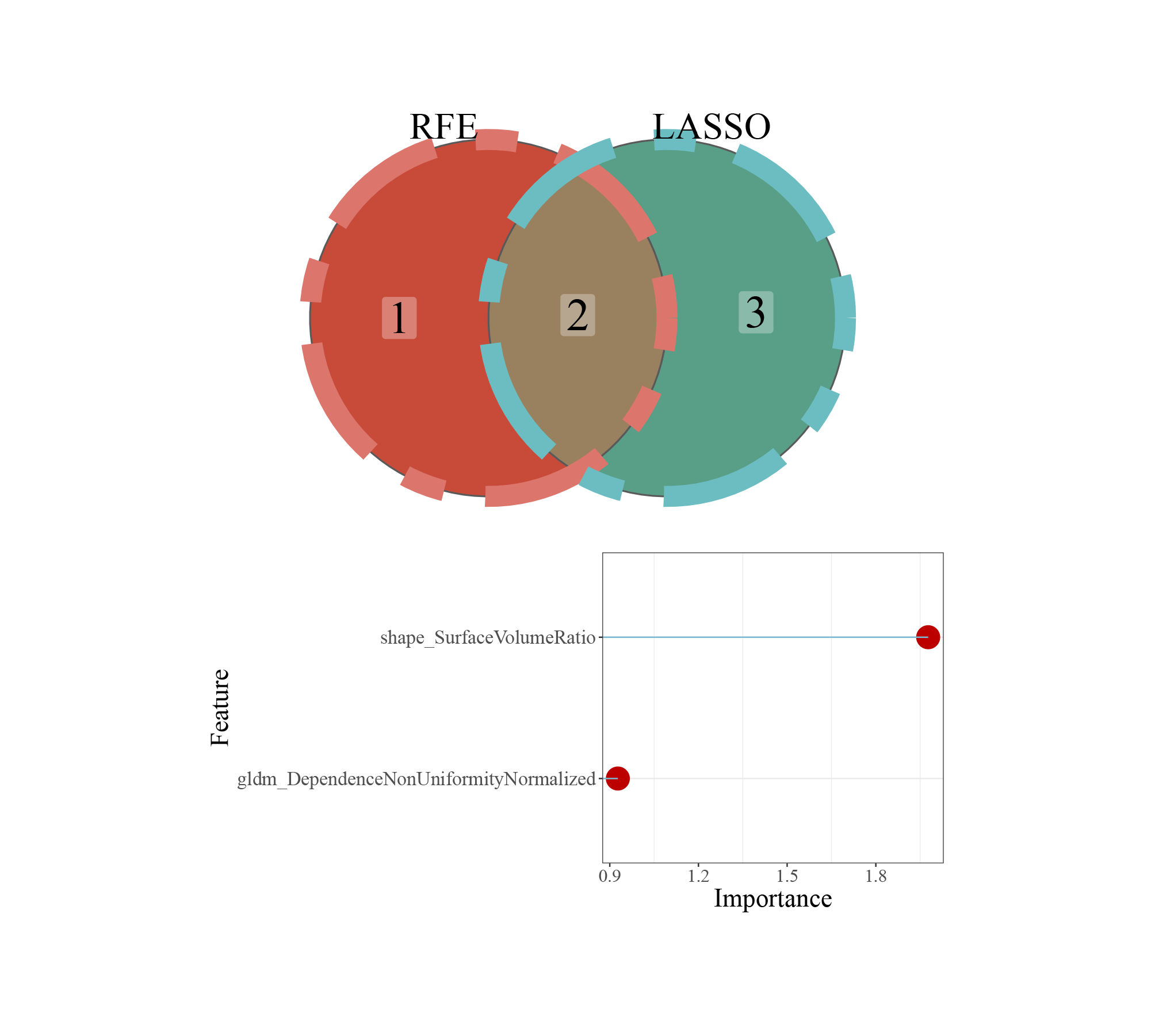
**

**Supplemental Fig.6：Assessment of the intersection-LR radiomic model for level prediction of CXCL9.** The training sets presented an accuracy of 0.684, a sensitivity of 0.5 , a specificity of 0.889, PPV of 0.833 , NPV of 0.615, and Brier score of 0.204; The validation cohort showed an accuracy of 0.702, a sensitivity of 0.667, a specificity of 0.741, PPV of 0.741, NPV of 0.667, and Brier score of 0.211. In the training sets, the ROC-AUC (**A**) was 0.723 and the PR curve (**C**) showed AUC values of 0.740. In the validation cohort, the ROC curve (**B**) showed AUC values of 0.715 (95% CI: 0.579-0.851). The DeLong test between the cross-validation AUCs did not show a significant difference between the results, indicating a good model fit (P = 0.928). Calibration curves (**D**) calculated and plotted via Hosmer-Lemeshow test revealed a good consistence between predictive model and the actual gene levels (P = 0.527). As shown in the DCA (**E**), the intersection-LR radiomic model, with the maximum net benefit, showed a threshold probability of 0-0.76. High CXCL9 expression was with greater discrimination in their probability estimates in the CXCL9-high group ( P < 0.05 )(**F**).


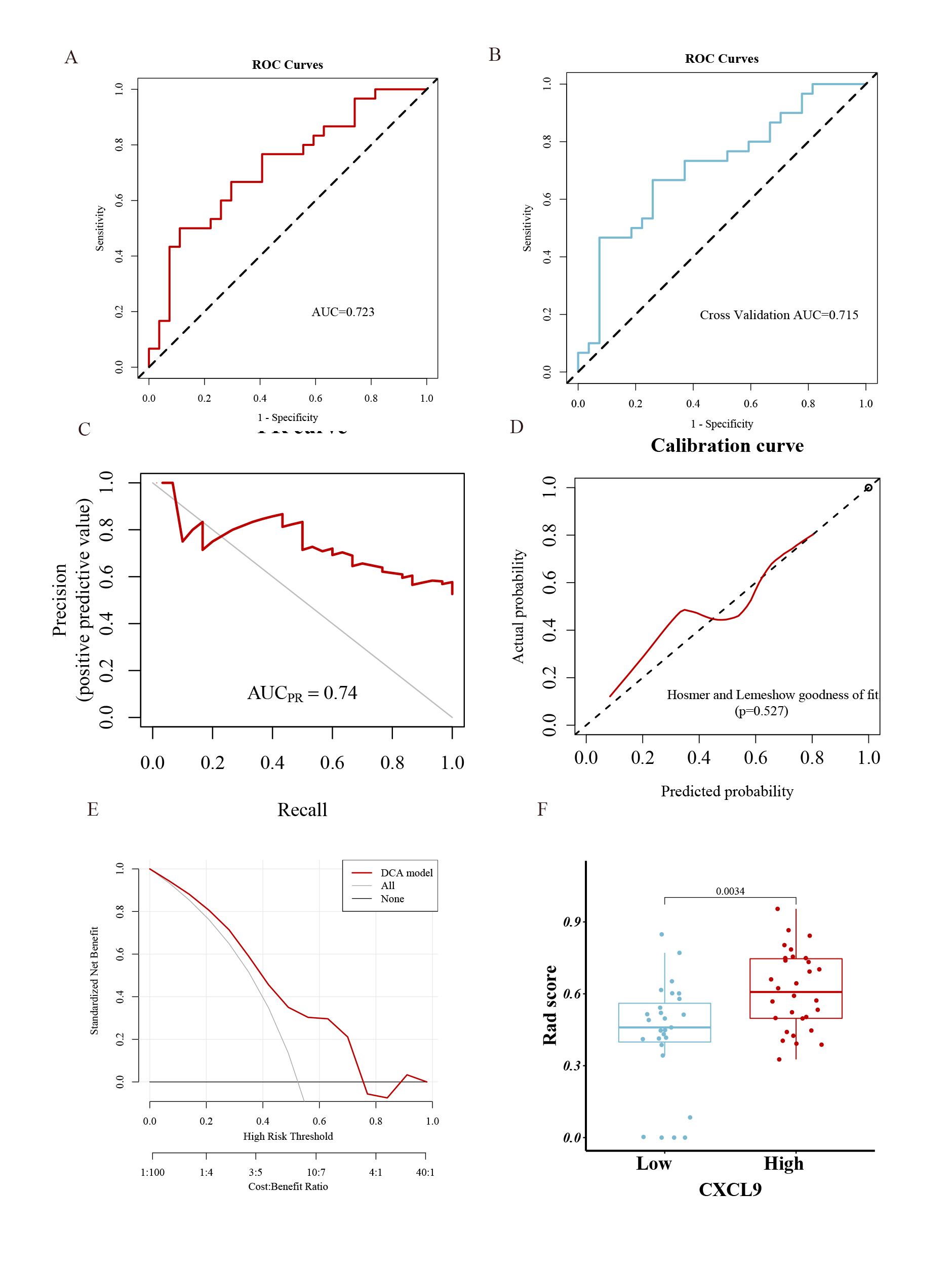


**Supplemental Table 4: Comparison of the AUC values between the radiomic models.**

|  | **RFE vs LASSO** | **RFE vs RFE_LASSO** | **LASSO vs RFE_LASSO** |
| --- | --- | --- | --- |
| train_p | 0.59 | 0.18 | 0.17 |
| vali_p | 0.86 | 0.64 | 0.77 |
